# Engineering Antigen-Specific T Cells from Genetically Modified Human Hematopoietic Stem Cells in Immunodeficient Mice

**DOI:** 10.1371/journal.pone.0008208

**Published:** 2009-12-07

**Authors:** Scott G. Kitchen, Michael Bennett, Zoran Galić, Joanne Kim, Qing Xu, Alan Young, Alexis Lieberman, Aviva Joseph, Harris Goldstein, Hwee Ng, Otto Yang, Jerome A. Zack

**Affiliations:** 1 Division of Hematology-Oncology, The David Geffen School of Medicine at University of California Los Angeles, Los Angeles, California, United States of America; 2 Division of Infectious Diseases, Department of Medicine, The David Geffen School of Medicine at University of California Los Angeles, Los Angeles, California, United States of America; 3 Department of Microbiology, Immunology, and Molecular Genetics, and The UCLA AIDS Institute, The David Geffen School of Medicine at University of California Los Angeles, Los Angeles, California, United States of America; 4 Departments of Pediatrics and Microbiology & Immunology, Albert Einstein College of Medicine, Bronx, New York, United States of America; New York University, United States of America

## Abstract

There is a desperate need for effective therapies to fight chronic viral infections. The immune response is normally fastidious at controlling the majority of viral infections and a therapeutic strategy aimed at reestablishing immune control represents a potentially powerful approach towards treating persistent viral infections. We examined the potential of genetically programming human hematopoietic stem cells to generate mature CD8+ cytotoxic T lymphocytes that express a molecularly cloned, “transgenic” human anti-HIV T cell receptor (TCR). Anti-HIV TCR transduction of human hematopoietic stem cells directed the maturation of a large population of polyfunctional, HIV-specific CD8+ cells capable of recognizing and killing viral antigen-presenting cells. Thus, through this proof-of-concept we propose that genetic engineering of human hematopoietic stem cells will allow the tailoring of effector T cell responses to fight HIV infection or other diseases that are characterized by the loss of immune control.

## Introduction

The human immune system is normally highly effective in managing exposure to the constant array of environmental antigens encountered. However, there are many instances where the immune response is ineffective in clearing infection or tumors. T cell responses, particularly cytotoxic T lymphocyte (CTL) responses, are critical in controlling viral infection or abnormal cellular growth and the failure of this response is a large factor in the inability to control these conditions[1]. Many current approaches toward treating a variety of diseases, particularly persistent diseases such as cancers or chronic viral infections, focus on the correction of defects in cellular function. Gene therapy approaches have been utilized to protect cells from infection, correct genetic defects, and enhance immune responses; however, gene-based approaches to directly enhance human cellular immune responses are relatively unexplored.

Previous studies utilizing standard gene transfer technologies have demonstrated that cloned, antigen–specific T cell receptors (TCRs) can be used to target polyclonal mature peripheral blood derived CD8+ T cells towards viral and cancer antigens [2]–[8]. This approach has been utilized in safely treating melanoma-afflicted individuals by “redirecting” peripheral CD8+ T cells following transduction with a vector containing an antigen specific TCR against the MART-1 antigen [9], [10]. The introduction of tumor antigen-specific cells in this instance resulted in successful tumor regression in some treated individuals [9]. However, while cells carrying the transgene in this study appeared to be long-lived, extensive ex vivo manipulation resulted in intrinsic functional defects [9]. In addition, these transduced cells also expressed endogenous TCRs and the introduction of a second TCR bypasses thymic selection and could result in auto-reactivity through cross-pairing of TCR chains or circumventing peripheral tolerance. Thus, the use of a gene therapy approach utilizing hematopoietic stem cells (HSCs) that produces functional, naive CD8+ T cells carrying a single desired antigen-specific TCR, could allow long-term engraftment, continuous generation of new effector cells, and a more efficient response through natural immune mechanisms.

Transgenic mice carrying murine TCR transgenes for a variety of antigens have been developed and are a common tool in examining cellular differentiation and function[11], [12]. Yang and Baltimore recently showed that cloned mouse TCRs introduced into murine HSCs can differentiate into antigen-specific T cells [13], [14]. Investigators have demonstrated the expression of introduced TCRs following differentiation of human progenitor cells on mouse stromal cell lines expressing the Delta-like 1 molecule [15], [16]. However, the resultant TCR-expressing cells in these studies did not undergo normal positive and negative selection events that a developing T cell would in the human thymus. Furthermore these studies did not address whether a disease fighting TCR can direct human T cell differentiation *in vivo* following genetic modification of human HSCs.

In the current study, we examined genetic HSC modification to produce antigen-specific T cell immunity. To determine if functional human CD8+ T cells expressing a transgenic antigen-specific human TCR can be derived from genetically modified human HSC, we utilized a human leukocyte antigen (HLA)-A*0201 restricted TCR specific for the highly conserved HIV p17 gag peptide SLYNTVATL (SL9) derived from CD8+ T cells from an infected individual. In addition, we utilized the chimeric severe combined immunodeficient mouse/human (SCID-hu) system in which mice are transplanted with human fetal thymus and liver under the renal capsule, forming a conjoint human organ that phenotypically and functionally recapitulates human thymopoiesis within the mouse [17], [18]. This provides the optimal environment for the study of human T cell differentiation within a surrogate host without having to directly involve human subjects. We and others have shown that injection of exogenous, allogeneic CD34+ HSC progenitors into sublethally irradiated SCID-hu mice results in engraftment and *de novo* differentiation of the exogenous cells into mature T lineage cells [19], [20]. We now demonstrate the ability to effectively transduce HSCs with an HIV-specific TCR, leading to the development of a large population of mature, functional human T cells able to specifically kill cells presenting viral peptide. This establishes a unique system to examine human TCR transgenic HSC development and facilitates the use of antigen-specific TCRs to enhance human T cell immunity and allows the close examination of the mechanisms of human T cell development and thymic selection.

## Methods

### Ethics Statement

Peripheral blood from HIV+ and HIV- individuals was obtained at the University of California, Los Angeles in accordance with UCLA Institutional Review Board (IRB) approved protocols under written informed consent (using an IRB-approved written consent form) by Dr. Yang and the UCLA Center for AIDS Research Virology Laboratory and was distributed for this study without personal identifying information. Resulting molecularly cloned TCRs were synthesized based on sequence information from these samples, and subsequent use did not require additional IRB approvals. Human fetal tissue was purchased from Advanced Biosciences Resources and was obtained without identifying information and thus did not require IRB approval for its use. All of the animal research described in this manuscript was performed under approval of the UCLA Animal Research Committee in accordance to all federal, state, and local guidelines.

### Cloning of the HIV-1 Gag Protein, SL9 Epitope-Specific TCR

The TCR was initially isolated from the SL9-specific CTL clone 1.9, which was obtained under a UCLA IRB approved protocol, using the 5′ rapid amplification of cDNA ends (RACE) method [21]. Using overlapping PCR, the cloned TCRα and TCRβ were then joined by a picornavirus-like 2A “self-cleaving” peptide. The short 18 amino acid 2A sequence which separates the TCRα and TCRβ results in equimolar expression of the TCRα and TCRβ via a? “ribosomal skip” mechanism [22]. The TCRα-2A-TCRβ gene was cloned into the pCCL.PPT.hPGK.tcr.IRES.eGFP lentiviral vector under control of the human phosphoglycerate kinase promoter (hPGK), followed by an internal ribosomal entry site (IRES) upstream of the GFP gene [5]. The 1.9 TCR sequence was then synthetically codon optimized using sequence information based on the above clone for maximum expression in human cells and was re-inserted into the pCCL.PPT.hPGK.tcr.IRES.eGFP lentiviral vector [23].

### Lentiviral Vector Production

Infectious, replication incompetent lentivirus was produced using the Invitrogen ViraPower Lentiviral Expression System with 293FT cells and the Lipofectamine 2000 reagent (Invitrogen, Carlsbad, CA). Briefly, 293FT cells were co-transfected simultaneously with the TCR containing pCCL.PPT.hPGK.1.9.IRES.eGFP plasmid, the pCMV-ΔR8.2-Δvpr packaging construct, and the pCMV-VSV-G vesicular stomatitis virus glycoprotein (VSV-G) expressing plasmid. Control eGFP expressing vectors were derived from the pCCL.PPT.hPGK.eGFP construct (in place of the TCR containing construct) in a similar manner. Supernatant was harvested from transfected 293FT cells 24 hours following transfection, filtered using a 0.22 µm sterile filter, and concentrated by centrifugation using a Beckman SW32 rotor at 30,000 rpm. Pellets were resuspended in phosphate buffered saline overnight at 4°C and frozen in aliquots at −80°C until used. Titration of the vector stocks and specificity of the TCR was performed on Jurkat T cells by limiting dilution and flow cytometry for eGFP and SL9-specific tetramer staining. Alternatively, the pCCL.PPT.hPGK.1803.IRES.eGFP plasmid [5] containing the 1803 TCR was used in some experiments in place of the 1.9-containing plasmid.

### TCR-Containing Vector Transduction of CD8+ PBMC

CD8+ T cells were purified from fresh human PBMC using the EasySep CD8+ T cell enrichment Kit (StemCell Technologies) and were stimulated with anti-CD3 and irradiated allogeneic PBMCs for 4 days. Cells were then transduced with the lentiviral vector overnight and incubated for 2 more days. IFN-γ production was then measured by enzyme-linked immunospot (ELISPOT) using IFN-γ specific capture and detection antibodies (Pharmingen)[24] following incubation with irradiated (3,500 rads), peptide-coated (0.1 µg/ml) 174xCEM.T1 (T1) cells (a professional antigen presenting cell line that express high levels of HLA-A*0201) cells or non-peptide coated cells. SL9 (SLYNTVATL) peptide was purchased from Anaspec Inc. Results were read by an ImmunoSpot Analyzer (Cellular Technology Ltd.).

### Isolation of CD34+ HSCs

Fresh human fetal liver was obtained by Advanced Biosciences Resources Inc. or at local sties under appropriate UCLA internal review board guidelines (Alameda, CA) and homogenized by slicing the tissue into pieces with a scalpel and passing it into a 12 ml syringe fitted with a 16 gauge blunt needle several times. The tissue was then digested with collagenase, hyaluronidase, DNase in Iscoves Modified Dulbeccos Medium (IMDM) for 90 minutes at 37°. Cells were then underlayed by Ficoll and spun 2400×g for 20 minutes and the interface was collected. Cells were repeatedly washed and placed into culture overnight at a concentration of 4×10^6^ cell/ml of RPMI 10% FCS and 0.44 mg/ml Piptazo (Piperacillin and Tazobactam). CD34+ cells were then isolated utilizing direct human CD34+ cell isolation Kit (Miltenyi) followed by magnetic activated cell sorting by an AutoMACS (Miltenyi) apparatus. Purified cells were then immediately viably frozen in 90% FCS, 10% DMSO and kept in liquid nitrogen storage until use.

### Transduction of CD34+ HSCs

Purified CD34+ cells were thawed from liquid nitrogen storage, washed and resuspended in 2% human serum albumin (HSA) containing Yssel's medium and placed into a 6-well plate coated with 20 µg/ml retronectin (Takara Bio, Inc.) with the lentiviral vector at a multiplicity of infection (MOI) of 1 overnight at 37°C. Cells were then washed, placed in plain minimal essential medium, and then injected into human thymic implants in SCID-hu mice. To determine transduction efficiency, 1×10^5^ cells were removed prior to injection and cultured in IMDM containing 20%FCS, 50 ng/ml of IL-3, IL-6, and SCF for 3 days. eGFP expression was then assessed by flow cytometry.

### SCID-hu Mice

Human fetal liver and thymus SCID-hu mice were constructed as previously described [18], [25]. Mice were irradiated with 300 rads from a colbalt-60 source to clear endogenous thymocytes from transplanted human tissue prior to implantation of 1×10^6^ human HSCs per implant. To assess human tissue, mice were surgically biopsied utilizing survival surgical techniques as described [25].

### Flow Cytometry

Cells were phenotypically analyzed using monoclonal antibodies specific for human CD3, CD45, CD4, CD8 (Coulter) and CD27, CD45RA, CCR7, and HLA-DR (eBioscience) conjugated to phycoerythrin (PE), electron coupled dye (ECD), allophycocyanin, PE-cychrome5 (PC5), or PE-cychrome 7 (PC7). Tetramer expressing cells were identified utilizing MHC Class I tetramer containing the SL9 peptide conjugated to PE (Coulter). Cells were assessed either by a Coulter FC500 instrument or a Becton Dickinson LSR2 instrument and results were analyzed FlowJo software.

### Measurement of Functional Transgenic SL-9 Specific TCR Activity

To assess the ability of thymocytes containing the transgenic TCR to express IFN-γ in response to peptide specific stimulation, following biopsy and homogenization of thymic tissue, cells were placed in culture at a concentration of 1×10^6^/ml in the presence of irradiated (3,500 rads) human T1 cells pretreated with 0.1 µg/ml SL9 peptide and 20 units/ml of IL-2 in RPMI 10% FCS. Cells were then examined for IFN-γ production by ELISPOT as described above.

CTL lytic ability was assessed by stimulating biopsied thymocytes with 2×10^6^/ml irradiated B (Patient 1, HLA-A*0201+) cells pre-coated with SL9 peptide (1.0 µg/ml) and 2×10^6^/ml irradiated allogeneic PBMC for 1 week in the presence of 50 units/ml of IL-2 (Roche). A fraction of cells was removed and analyzed by polychromatic flow cytometry. The remainder of cells were then placed in a standard ^51^chromium release assay at an effector to target cell ratio of 10∶1 for 5 hours using irradiated (3,500 rads) 174xCEM.T2 (T2) cells (which are also HLA-A*0201+) coated with SL9 peptide (1.0 µg/ml) as targets.

## Results

### Isolation and Cloning of HIV-Specific TCR

**Figure 1:**
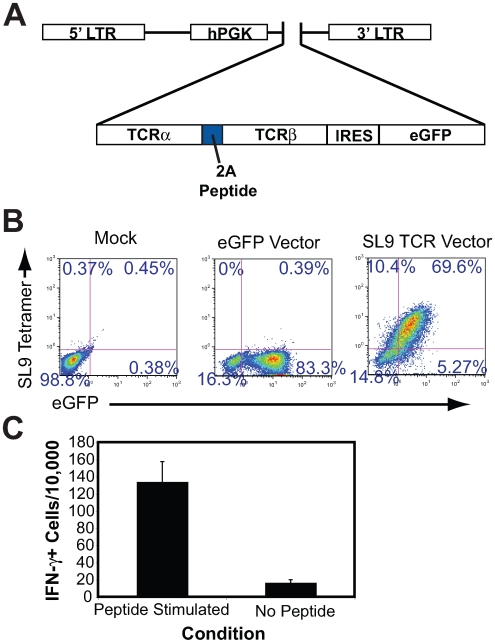
Cloning and functional expression of an HIV gag SL9 epitope specific TCR. (A). Diagram of the pCCL.PPT.hPGK.tcr1.9.IRES.eGFP lentiviral vector expressing the SL9 specific TCR utilized to genetically manipulate CD34+ cells. LTR, long terminal repeat; hPGK, human phosphoglycerate kinase promoter element; TCRα, alpha chain; 2A peptide, picornavirus 2A self cleaving peptide; TCRβ, beta chain; IRES, internal ribosomal entry site; eGFP, enhanced green fluorescent protein. (B). Transduced Jurkat T cells were analyzed by flow cytometry for expression eGFP and SL9-specific TCR by SL9 tetramer staining 2 days following transduction with the TCR containing lentiviral vector. The numbers represent the frequency of cells expressing the transgenic TCR and eGFP. (C). Purified CD8+ T cells were transduced with the lentiviral vector containing the SL9-specific TCR and assessed by ELISPOT analysis for their ability to express IFN-γ in response to peptide specific stimulation (left) or no peptide treatment (right). The numbers indicate the average number of cells expressing IFN-γ per 10,000 total cells (n = 7 total replicate cultures).

To test the feasibility of directed T cell development following transduction of HSC with a human TCR, we utilized a human leukocyte antigen (HLA)-A*0201 restricted TCR specific for the highly conserved HIV p17 gag peptide SLYNTVATL (SL9) derived from CD8+ T cells from an infected individual. To isolate this TCR, peripheral blood mononuclear cells (PBMCs) from an HIV infected individual were selected by culturing cells in the presence of the SL9 peptide [26]–[28]. Full-length α and β TCR genes from a CTL clone (termed 1.9) specific for SL9 were amplified using reverse transcriptase polymerase chain reaction (PCR). The sequence was then optimized for expression and subsequently cloned into a lentiviral vector under the control of a human phosphoglycerate kinase (hPGK) promoter element. The unique TCR α and β genes, specific for viral antigen, were placed into this vector separated with a 2A self-cleaving peptide (or “skip” peptide) where the two chains are expressed together with high efficiency [29](Figure 1A). This vector further contains an internal ribosomal entry site (IRES) to allow expression of the enhanced green fluorescent protein (eGFP) marker gene. To initially assess the antigen specificity and expression of the 1.9 TCR, we transduced Jurkat T cells and observed efficient and linked expression of the 1.9 TCR and eGFP (Figure 1B). To assess the functional competence of this TCR to induce cytokine responses, mature human peripheral blood mononuclear cell (PBMC)-derived CD8+ T cells were transduced and stimulated with an irradiated SL9 peptide-pulsed HLA-A*0201+ professional antigen presenting cell line. This effectively induced IFN-γ production and demonstrates that the expression of this TCR in CD8+ T cells can allow antigen-specific functional responses (Figure 1C). In all, this demonstrates the isolation of a functional, SL9 reactive TCR and provided the impetus towards further examination of transgenic TCR expression in a hematopoietic differentiation system.

### Differentiation of Human TCR Transgenic Cells *In Vivo*

**Figure 2:**
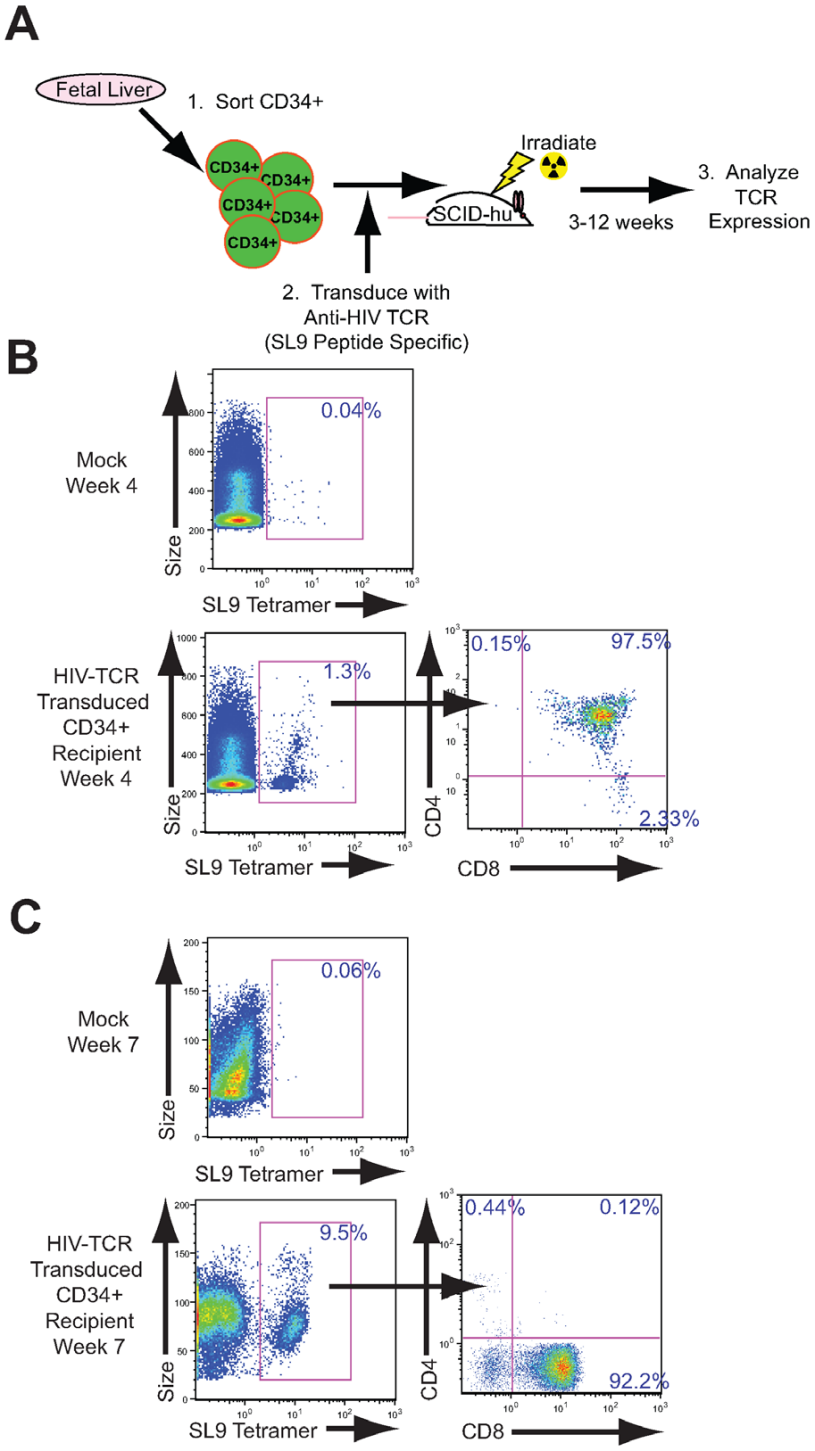
Genetic manipulation of HSCs and generation of thymocytes. (A). Schematic illustrating the isolation of CD34+ cells from fetal liver by cell sorting (1), transduction of progenitor cells with lentiviral vector containing the SL9-specific TCR (2), and implantation of these cells in the thymic tissue implanted in sub-lethally irradiated SCID-hu mice. Following 3–12 weeks to allow T cell development to occur from HSCs, TCR expression was analyzed and functional assays performed (3). At four (B) and seven (C) weeks following implantation with TCR transduced HSCs, human thymic tissue was biopsied and cells were analyzed by flow cytometry for cell size (forward scatter—denoted “Size”) and SL9 specific tetramer staining. Mock treated mice (top panels) and mice receiving TCR transduced cells (bottom panels) are indicated. The numbers on the left panels illustrate total SL9 tetramer staining cells. SL9-tetramer+ cells were gated and the frequency of cells expressing CD4 and/or CD8 are provided in the right panels.

**Figure 3:**
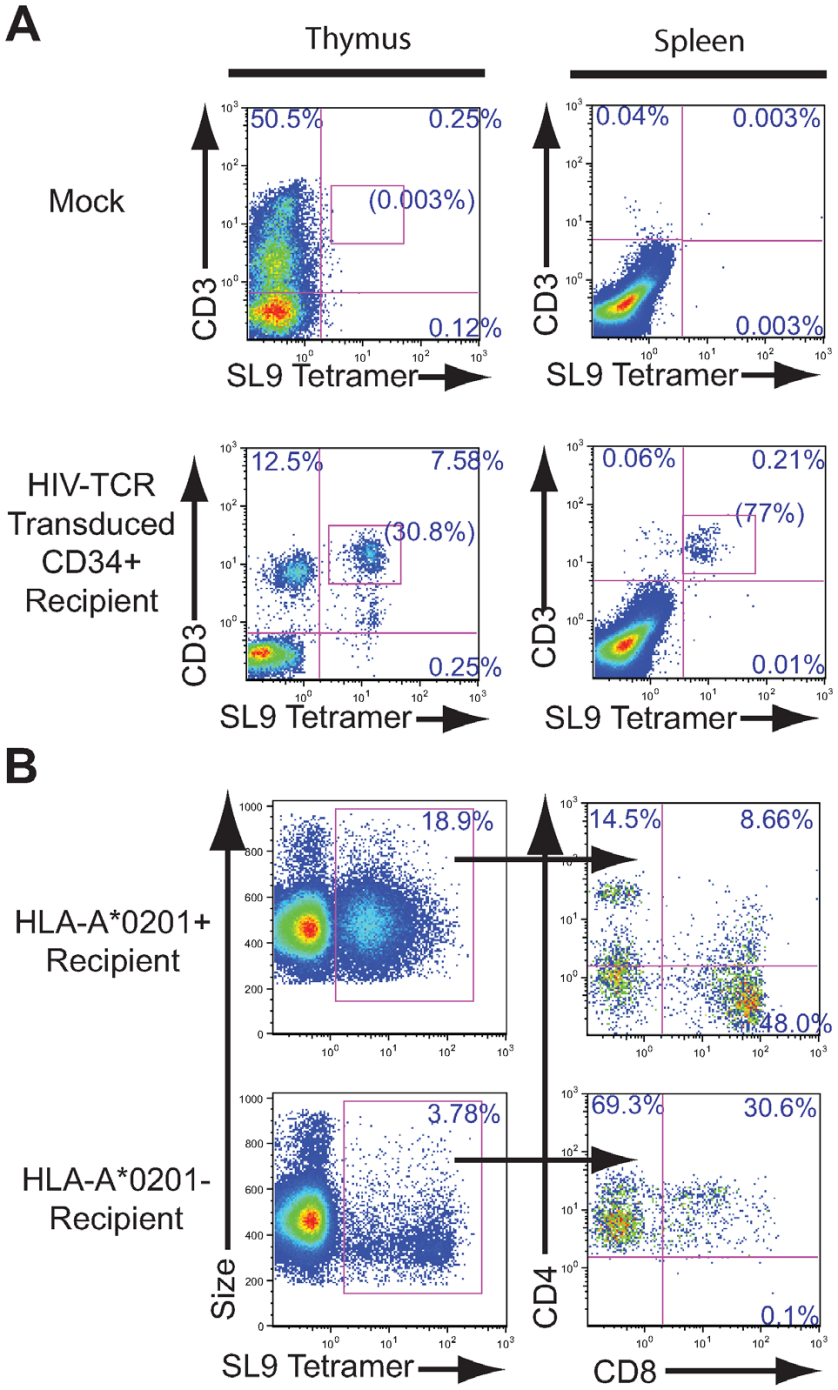
Characterization of SL9-specific TCR transduced HSC development into T cells. (A) Mock treated mice (upper row) and mice receiving HSCs transduced with the HIV SL9-specific TCR (lower row) were analyzed 7 weeks following transplantation for CD3 and SL9-specific TCR expression by tetramer staining of cells from the thymus (left panels) or spleen (right panels). The frequency of CD3+ and SL9-tetramer+ cells is provided and the values inside the parentheses correspond to the percentage of tetramer positive cells in the human T cell (CD3+) populations. (B) Fetal liver derived CD34+ HSCs transduced with the SL9-TCR containing lentiviral vector were implanted into mice containing either HLA-A*0201+ thymic tissue (top panels) or into mice containing HLA-A*0201- thymic tissue (bottom panels) and the frequency of SL-9 tetramer+ cells assessed 6 weeks following implantation. Size (forward scatter) versus tetramer staining is presented in the left panels and the values inside the parentheses correspond to the percentage of tetramer positive cells. Tetramer expressing cells in the indicated gate were assessed for CD4 and CD8 expression (right panels) and the frequencies of cells expressing each marker are provided.

To determine if functional antigen-specific T cells can be derived from human TCR -transduced HSCs, CD34+ HSCs were purified from fetal liver and transduced with the cloned anti-HIV 1.9 human TCR. Transduction efficiency was typically 60–80% of CD34+ cells (data not shown). These cells (1×10^6^ HSCs per mouse) were then injected directly into HLA-A*0201+ human thymic implants in sub-lethally irradiated SCID-hu mice. The irradiation was performed to deplete endogenous thymocytes to clear space within the thymic tissue for the newly implanted cells. Thymocytes were allowed to develop from transduced CD34+ HSCs following implantation into the human tissue and cells containing the transgenic TCR were analyzed following biopsy of the thymic tissue (Figure 2A). In the thymus, from CD34+ HSCs it typically take 1–3 weeks to begin the development of immature CD34-CD4+CD8+ thymocytes and an additional 2–4 weeks to begin to see the appearance of mature CD34-CD4-CD8+ or CD34-CD4+CD8- T cells. In these studies, within 4 weeks following transplantation of the transduced hematopoietic progenitor cells, a distinct population of immature CD4+CD8+ thymocytes expressing the transgenic SL9-specific TCR was observed (Figure 2B). Interestingly, even at this early time point mature CD4-CD8+ cells were beginning to emerge, indicating that differentiation into HIV specific cells can occur relatively rapidly following transplantation of TCR transduced cells. Within 7 weeks following implantation of 1.9 TCR transduced HSCs, a substantial frequency of thymocytes expressed the transgenic TCR and the majority of these cells were mature CD4-CD8+ thymocytes (Figure 2C) indicating that these cells were undergoing appropriate lineage commitment. Importantly, we further observed that these transgenic SL9-specific TCR-containing T cells were found in the spleens of reconstituted mice (Figure 3A), indicating that cells expressing the transgenic TCR can progress through thymic differentiation and home to peripheral lymphoid tissues. In subsequent studies, we also observed similar results utilizing a different, previously characterized TCR (the 1803 clone [5]) specific to the SL9 peptide, indicating that cells containing a different TCR derived from a different patient and using different TCR alpha and beta genes differentiated into thymocytes in a similar manner (data not shown).

For a hematopoietic cell to successfully become a mature, functional antigen-specific CD8+ T cell, the TCR bearing cell must undergo positive and negative selection in the thymus in the context of specific HLA Class I molecules. In the absence of the proper signal from the appropriate restricting molecule, the developing T cell will fail to become a mature CD8+ T cell. To examine the ability of these TCR transgenic cells to develop in the presence or absence of the exact SL9 peptide restricted HLA molecule (HLA-A*0201), we injected SL9-specific TCR-transduced CD34+ cells into mice implanted either with HLA-A*0201+ or HLA-A*0201- thymic tissue. Mice implanted with HLA-A*0201+ thymic tissue displayed differentiation of transduced HSCs into mature CD4-CD8+ T cells that expressed the transgenic SL9 specific TCR whereas mice implanted with HLA-A*0201- thymic tissue initially developed immature CD4dimCD8- and CD4+CD8+ thymocytes[30], but no mature CD4-CD8+ cells were observed (Figure 3B). These results indicate that the appropriate restricting elements of the recipient tissue are required for the proper development of human transgenic antiviral specific TCR-containing cells into mature thymocytes and T cells. We noted development of CD8+ tetramer+ cells in the thymic tissues derived from 6 out of 7 HLA-A*0201+ fetal donors, which would express a variety of other MHC Class I molecules. This suggests that the developing cells that express this particular TCR, and are positively selected on HLA-A*0201, survive the negative selection process in the context of a wide array of other HLA molecules.

### HSC-Derived Virus-Specific T Cells Are Functional and Possess Antiviral Activity

**Figure 4:**
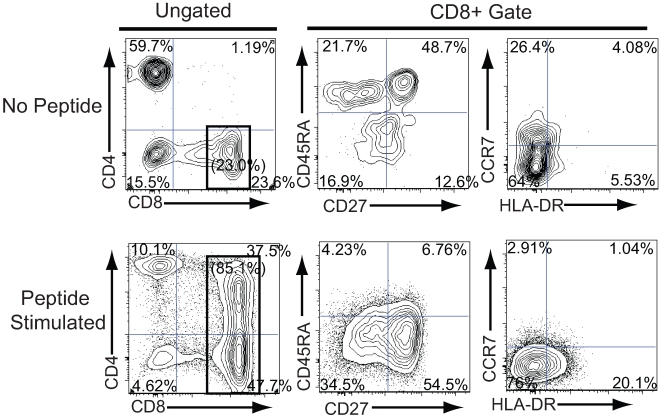
Phenotypic CD8+ T cell differentiation following SL-9 peptide-specific stimulation of transgenic TCR expressing thymocytes. Thy/Liv implants from mice receiving transduced CD34+ cells were biopsied following development of mature TCR expressing cells and isolated thymocytes cultured in the presence of irradiated feeder cells, an irradiated HLA-A*0201+ B cell line and without (top row) or with (bottom row) the SL9 peptide for one week. Cells were removed and analyzed utilizing polychromatic flow cytometry for the indicated markers. Due to down regulation of TCR following peptide-specific stimulation, lentiviral vector expressing cells were identified by expression of eGFP and were gated and analyzed for CD4 versus CD8 expression (left panels). CD8 T cells were then examined using the indicated gate for expression of differentiation markers (middle and right panels). Percentages indicate cells staining in each quadrant and the numbers in parenthesis indicate the percentage of cells within the CD8+ gate.

**Figure 5:**
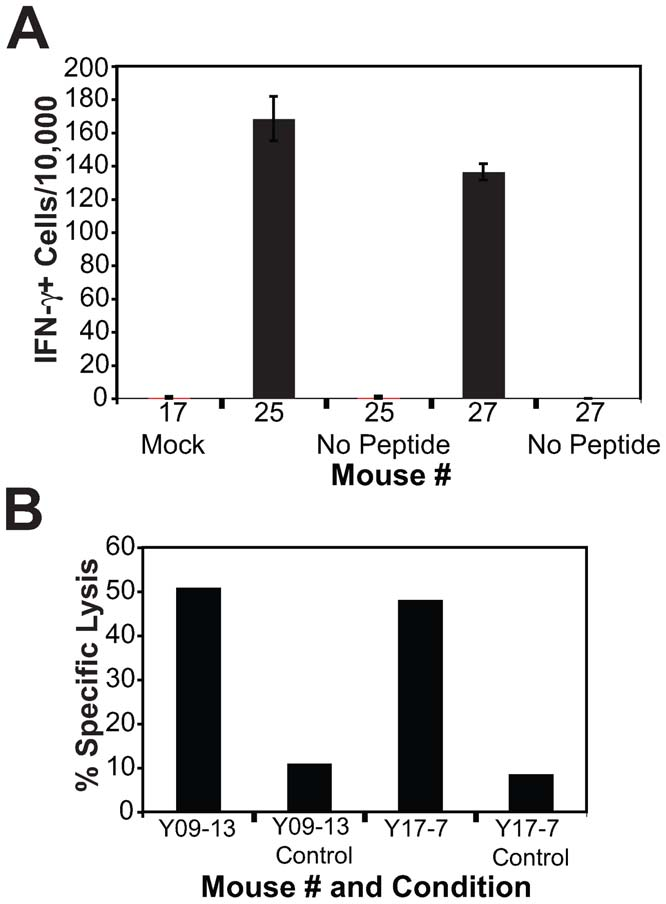
Functional responses of HIV-specific TCR transgenic T cells. (A) Thymic tissues from 2 mice receiving SL9-specific TCR transduced HSCs and 1 mock-treated mouse were biopsied 7 weeks following introduction of HSCs and placed into culture with SL9 peptide coated antigen presenting cells for 1 week to allow differentiation from antigen naïve to effector cells. Effector cells were then stimulated with SL9 peptide or medium alone (no peptide) and IFN-γ production was measured by ELISPOT. (B) Cells from SCID-hu mice receiving SL9-specific TCR transduced HSCs were obtained by biopsy following differentiation into thymocytes and activated in culture in the presence of an irradiated SL9-peptide coated HLA-A*0201+ B cell line and allogeneic PBMC feeder cells. Cells from mouse numbers Y09-13 and Y17-7 were then placed in a standard ^51^chromium release assay utilizing SL9 peptide coated T2 cells or untreated T2 cells as a control. Graph shows the specific lytic activity of cells at an effector to target cell ratio of 10∶1.

To determine the functional capacity of the newly developed viral antigen-specific TCR-containing T cells, thymic tissue from SCID-hu mice receiving the SL9 specific TCR transduced HSCs was biopsied 7 weeks after injection. Since these antigen-naïve cells lack functional activity prior to cellular activation, the naïve cells were stimulated with SL9 peptide-pulsed, irradiated HLA-A*0201+ cells. Peptide-specific stimulation of naïve, transgenic SL9-specific TCR expressing cells resulted in the normal phenotypic differentiation into cells possessing an effector phenotype [31], [32](CD8+CD45RA-CD27+CCR7-)(Figure 4). In addition, these previously naïve, SL9-antigen specific cells induced the expression of the CD4 molecule following cellular activation, similar to that described by our laboratory and others' [33]-[37]. Following 1 week in culture, these effector SL-9 specific T cells produced significant levels of IFN-γ in response to peptide-specific stimulation (Figure 5A). To assess the cytolytic capacity of genetically engineered SL9-specific T cells, SL9-specific TCR-transduced T cells were examined for their ability to lyse peptide labeled HLA-A*0201+ target cells following peptide-specific activation. These stimulated T cells significantly lysed peptide-labeled target cells in an antigen-specific manner (Figure 5B). Thus, thymocytes expressing the transgenic, SL9-specific TCR are functional in their ability to differentiate into effector T cells, release antiviral cytokines and kill antigen-expressing target cells. This demonstrates that this system can be utilized to closely examine human antigen-specific, naïve cellular responses.

## Discussion

The development of vaccine strategies, particularly therapeutic vaccine strategies, against many viruses that produce chronic infections in humans has proven to be difficult. In HIV-1 infection, the CD8+ T cell CTL response plays a crucial role in controlling viral replication in the infected individual [38], [39]. Inevitably, the CTL response fails and this loss is associated with an increase in viral load and a more rapid progression to AIDS [40], [41]. One method of augmenting CTL responses is to expand autologous antigen-specific CTLs ex vivo followed by the return of these cells into the affected individual. This has been shown to be effective in immunodeficient patients for the generation and enhancement of immunity to infection and control with cytomegalovirus (CMV) and Epstein-Barr virus (EBV)[42], [43]. While autologous HIV-specific CTLs placed in infected individuals have been shown to migrate to sites in the body of viral replication and retain some of their ability to respond to virally infected cells, this has not been shown to be effective in treating HIV infected individuals [5], [44]–[46]. In these HIV infected individuals, these adoptively transferred CTL persisted in the body for only a relatively short period of time and did not have a significant impact on viral replication[44]–[46]. These cells likely lack complete functional competence and the ability to properly respond to antigen and expand as a direct result of the effects of ongoing HIV infection and CTL clonal exhaustion prior to and following ex vivo expansion. *Ex vivo* expansion of these dysfunctional T cells is therefore insufficient to improve the antiviral CTL response. Thus, an alternative strategy to generate naïve antigen specific CTLs that would reconstitute immune function would be beneficial to controlling viral replication. Therefore, augmenting these CTL responses with virus-specific CTL could result in better immune control of viral replication and delay or prevent disease progression.

Generation of antigen-specific T cells from hematopoietic progenitor cells has the potential to generate long-term engraftment of specific immune cells through two different mechanisms: 1) the engraftment of hematopoietic stem cells and the production of progeny cells for extended periods of time, 2) the expansion of antigen reactive cells in the periphery and the differentiation of these cells into long-term memory cells. Our results indicate that introduction of a functional TCR into a hematopoietic progenitor cell can lead to the efficient generation of antigen-specific T cells with cytotoxic capabilities. This suggests that this approach could be useful clinically.

The ability of HIV to rapidly escape immune pressure would mandate the need for several TCRs specific for multiple viral epitopes. In the current studies, rather than seeing continuous production of immature thymocytes, we observed an extended wave of thymopoiesis culminating in the appearance of mature CD8+ thymocytes. This lack of long-term engraftment may reflect transduction of a more mature progenitor cell incapable of continuous self-renewal. Alternatively, these results may reflect the inability of these transduced stem cells to locate the correct hematopoietic niche in the SCID-hu model. Consistent with either of these mechanisms, we also observed transient reconstitution in this model using HSCs derived from embryonic sources [47]. Nonetheless, our studies provide proof of principle that this approach has strong merit.

Human stem cell gene therapy is a relatively new technology. While its use clinically at the current time is limited to a subset of diseases, its potential in treating multiple human diseases is immense. A relatively new approach is to genetically manipulate hematopoietic stem cells followed by re-infusion of these cells back into the patient. Our previous SCID-hu studies demonstrated that in the context of severe HIV-induced thymocyte depletion, human HSCs can properly differentiate into normal mature thymocytes provided that HIV replication is halted by antiretroviral therapy[48]–[50]. Gene therapy trials have effectively demonstrated that human stem cells can be transduced with a retroviral vector and subsequently form mature human T cells in adult subjects [51]. The recent completion of a large-scale phase 2 clinical gene therapy trial highlights the fact that this type of treatment can be used as a conventional therapeutic approach for people with HIV or a variety of diseases [52]. In all, our data demonstrate that HSC transduction with a human viral antigen-specific TCR can be utilized to generate antigen-specific CTL. Our data strongly suggest that this strategy should be pursued as an effective therapy to combat viral infection in humans.
